# Research and experiment on the trenching performance of orchard trenching device

**DOI:** 10.1038/s41598-023-46278-7

**Published:** 2023-11-02

**Authors:** Chen Ma, He Wei Meng, Jian Zhang, Cong Zhang, Ying Zhao, Li Hong Wang

**Affiliations:** 1 College of Engineering and Technology, Southwest University, Chongqing, 400700 China; 2 College of Mechanical and Electrical Engineering, Shihezi University, Shihezi, 832000 China; 3 Ministry of Education Engineering Research Centre for Mechanization of Oasis Specialty Cash Crop Produc, Shihezi, 832000 China; 4 Mechanical and Electrical Engineering College, Hainan University, Haikou, 570100 China

**Keywords:** Scientific data, Mathematics and computing, Software

## Abstract

Aiming at the mismatch between the cutter combination of the furrowing device and the operating parameters, and resulting in low quality of furrowing and other problems, the theoretical analysis of the furrowing cutting operation is carried out and the influence law of the furrowing parameters on the trajectory, performance and quality of the furrowing movement is obtained. The influence of trenching parameters on trenching trajectory, performance and quality was obtained. The response surface method was applied to design and carry out field experiments. With the increase of cutter head speed and forward speed, the width and depth of trenching and the thickness of floating soil at the bottom of trenching decreased first and then increased, while the operation power consumption presented the increasing trend gradually. The optimization model of trenching performance quality was constructed to obtain the optimal parameter combination of influencing factors. Field experiments were carried out to verify the optimization results. The optimisation results were verified through field tests, which showed that the average depth of furrowing was 472 mm, the width was 332 mm, the thickness of soil return was 134 mm, and the operating power consumption was 19.95 kW. The results showed that the average depth of furrowing was 472 mm, the width was 332 mm, and the thickness of soil return was 134 mm. The optimization model could meet the operation quality indexes, and provide a theoretical basis for the design of the disc subsection cutting trenching device to select the operation parameter combination required by low power consumption and deep trenching.

## Introduction

Fertilization is an important factor affecting fruit yield and quality^1^. Rational fertilization can promote the increase of fruit yield and income^2, 3^, and also improve soil fertility and environmental benefits of orchards. In the planting process of forest fruit, organic fertilizer is generally applied to improve the yield and quality of single fruit, the sugar-acid ratio and the content of soil organic matter. The application methods of organic fertilizer mainly include basal fertilizer, topdressing fertilizer and extra-root fertilizer^4–6^. Being affected by many factors, such as the deep application of organic fertilizer in orchards in Xinjiang -northwest China, planting patterns, working conditions, fertilization requirements of different varieties of forest and fruit, etc.^7–9^, the main application of organic fertilizer is artificial furrow fertilization. There are some problems, such as high labor intensity, low working efficiency, poor fertilizer uniformity and high cost. At present, plough-share, disc and chain-knife are usually used in mechanical furrow fertilization^10^, which has solved many problems faced by traditional artificial fertilization to some extent. However, due to the large amount of organic fertilizer applied in orchards, the narrow spacing among trees, and the different characteristics of soil materials, there are still some problems, such as unmatched structure of trenching equipment and planting pattern, insufficient trenching depth, high power consumption and multiple cutting of soil, etc. Relevant studies have shown that, due to the unreasonable selection of existing trenching devices and the mismatch with actual agronomic requirements, problems such as large power consumption, unstable trenching depth and thick floating soil at the bottom of ditch are more serious^11, 12^. Therefore, it is urgent to improve the adaptability of furrow device for deep application of organic fertilizer in orchards.

The disc trenching device has the advantages of low traction resistance, high operation efficiency, small structure size, and easy to cooperate with the fertilizer application device. It has gradually become the mainstream equipment for deep application of organic fertilizer trenching in orchards^13–15^. Based on the characteristics of fruit planting in Xinjiang, the disc trenching device is selected in this paper. At present, scholars at home and abroad have carried out a lot of theoretical analysis and experimental researches on the influence of the trenching parameters of the disc trenching device on the operation performance, quality, etc. Mouazen^16^ has analyzed the influence rule of the trenching device on the soil cutting process through the orthogonal test, and established the regression equation between the traction force and the soil moisture content, cutting depth; Ibrahmi^17^ established the functional equation between the type of rotary tillage device, geometric parameters and operating conditions on the force and soil disturbance, and verified the finite element simulation results of the interaction between soil and rotary tillage device on the soil tank test bench of sandy soil; Abo-Ennor^18^ constructed the structure model of rotary tilling tool-sand soil with the help of hydraulic conservative and carried out finite element simulation analysis. The results have shown that rotary tilling tool has a significant impact on the damage of predefined simulated soil; Zhang et al.^19^ designed a double-row trenching fertilization device with automatic adjustment of trenching depth according to the current situation of domestic orchard trenching fertilization machines, and verified that the stability of trenching depth and the uniformity of organic fertilizer distribution meet the requirements of orchard production through theoretical analysis and performance test; Ji et al.^20, 21^ used the strain gauge type torque sensor to carry out field verification test on the simulated rotary tillage tool. The test has shown that the tillage depth, cutter head speed, and unit forward speed have an impact on the operation power consumption during rotary tillage; Li et al.^22^ studied and analyzed the problems of serious soil back flowing and poor stability of trenching depth during the rotary trenching operation through simulation and field tests. The research has shown that the effect of the working parameters of the trenching device was trenching depth > the inclination of the surface > the rotation speed; Peng et al.^23^ carried out theoretical analysis and experimental verification on the problem of high success rate caused by the unreasonable selection of the operation mode and parameters of the disc trenching device, and carried out kinematic analysis on the forward and reverse direction of trenching, and established the adaptability equation of the forward and reverse direction to the depth of trenching; Liu et al.^24^ stablished a mathematical model between cutting, throwing soil and trenching device, and built a power consumption test device for trenching parts to find out the influence of the working and structural parameters of the 1 K–50 orchard trenching machine on the power consumption of the operation. Wang et al.^25^ designed a bionically coupled disc trenching device (BCDFO) and analyzed the soil disturbance, swelling rate and other index parameters through the discrete element simulation method of different structural parameters. From the above research status at home and abroad, it can be seen that the change of trenching operation parameters has a significant impact on the performance and quality of trenching. However, there are few studies on deep fertilization of organic fertilizer trenching in Xinjiang- northwest China orchards at present.

Therefore, based on the requirement of applying organic fertilizer, the planting pattern of fruit and the physical characteristics of soil in Xinjiang, China, the interaction characteristics between furrow device and soil will be analyzed in this paper by analyzing the interaction characteristics between trenching device and soil. With the goal of meeting the requirements of low energy consumption and deep trenching in the application of fruit organic fertilizer, and taking different trenching parameters as the relationship function of variables, the effects of furrow parameters on furrow performance and operation quality in the process of deep application of organic fertilizer will be studied to improve the operation quality of furrow equipment in Xinjiang.

## Materials and methods

### Theoretical analysis of trenching performance

#### Structure and working principle of trenching device

**Figure 1:**
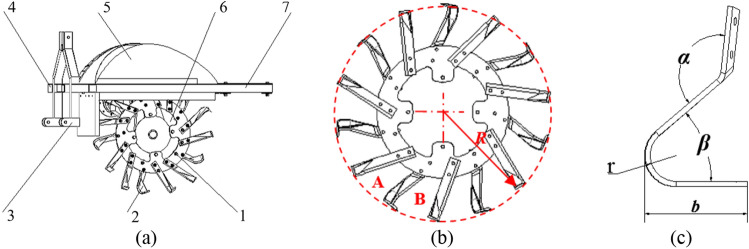
Schematic diagram of trenching device and trenching tool: 1. Cutterhead 2. Grooving knife 3. Traction frame 4. Cross beam 5. Shroud assembly 6. Cutterhead drive mechanism 7. Longitudinal beam. (**a**) Schematic diagram of the structure of the trencher; (**b**) Cutterhead assembly; (**c**) Structure diagram of trenching tool.

The trenching device (as shown in Fig. 1a) is mainly composed of a cutter head, a trenching cutter, a traction frame, a cross beam, a draft cover assembly, a cutter head driving mechanism and a longitudinal beam. The cutter head assembly comprises a trenching cutter A and a trenching cutter B, which were arranged in sequence by subsection spiral (A-B-A-B-…, each of A and B were divided into one group, 7 groups altogether, one group of cutting tools met the requirements of furrow agriculture with working width ≥ 250 mm), as shown in Fig. 1b. The main structure parameters of the groove cutting tool are: the radius of rotation r (according to the actual production requirements of the groove depth to choose a larger radius of rotation, so this design choose *R* = 600 mm), the angle of bending α (that is, the angle between the normal section and the line from the cutting edge to the center of the knife roll. If the bending angle is too small, the soil cutting angle of the cutting edge will be too large, increasing the soil cutting resistance and power consumption; if the bending angle is too large, the worn surface of the cutting tool will squeeze the uncultivated soil, and the force of the cutting tool will increase correspondingly, which will reduce the tool life, so choose 40° ~ 90°), the bending radius* R* (if the bending radius is too small, the bending arc is easy to clay, which will reduce the strength of the trenching tool in the bending, and shorten the service life; however, if the bending radius is too large, the unevenness of the trench bottom will be increased, and the power consumption will also be increased, so choose 50 mm^26^, the soil cutting angle *β* (if the cutting angle is too large, the trenching resistance will be increased, and the role of breaking soil will be reduced; but if the cutting angle is too small, the tool will easily wrap around the root system, which will reduce the working quality, so choose 130° ~ 180°), and the working width *b* (increasing the width can reduce the number of the tool on the roller, but if the bending radius is too large, it will affect the stiffness and the quality of the cutting tool, so choose *b* 90 ~ 190 mm) etc. The structure diagram of the trench cutter is shown in Fig. 1c.

The working principles are as follows: when the trenching device is cutting the soil, each group of trenching tools completes the required working width of trenching in turn, and ensures that each tool performs a cutting operation on the soil, so that each trenching tool can cut the soil in sections. The cut soil will be thrown up to the inside of the deflector with the rotation of the trenching tool, and fall to both sides of the trench under the effect of the deflector, thus completing the trenching operation.

#### Kinematic analysis of trenching operation

Research have shown that the structural parameters and working parameters of the trenching device have a certain impact on the trenching quality. In order to explore the influence rules of trenching operation, the kinematic analysis of trenching operation is carried out, and the movement track of the trenching tool tip point is constructed using the EDEM simulation software.

This section may be divided by subheadings. It should provide a concise and precise description of the experimental results, their interpretation, as well as the experimental conclusions that can be drawn.

#### Determination of simulation model parameters

Xinjiang forest fruits (jujube, apple, grape, etc.) application of organic fertilizer open furrow agronomic requirements (the depth of its open furrow shall not be less than 400 mm, open furrow width of not less than 250 mm)^27^, fertilizer used for fertilizer application is the second fermentation stable fertilizer. Xinjiang forest fruits are commonly planted in the bottom gravel grey desert loess^28^, whose soil characteristics are coarser soil texture, blocky structure, and greater firmness. Based on the physical properties of soil and the agronomy of fruit fertilization in Xinjiang, the cutting process simulation model of trenching device is established. The soil model and physical parameter selection use investigation results and soil sampling for later tests. According to the results of the experimental soil sampling, four shapes of soil particle models were established, namely, single sphere model, double sphere model, straight line three sphere model, and equilateral triangle-like three sphere particles, with a physical radius of 5 mm, which was chosen randomly when setting the physical radius of the particle plant particles in order to be closer to the soil authenticity. The Hysteretic Spring contact model was selected to model the soil channel using EDEM2020 software. In this paper, the parameters needed for simulation (as shown in Table 1) were determined by the combination of field soil measurement and literature^29–32^. Based on the requirements of fertilization agronomy and boundary, the model is set as a cuboid of 1500 mm × 2000 mm × 600 mm, and 1.8 × 10^6^ soil particles are generated to simulate the soil environment of trench cutting. In this paper, a time step of 1 × 10^−6^ s is set for the trenching device.

**Table 1:** Simulation parameters.

Objects	Attributes	Value
Soil granules	Poisson's ratio	0.26
	Shear modulus (Pa)	2 × 10^7^
	Density(Kg/m^3^)	2650
Disk(60Si2Mn)	Poisson's ratio	0.3
	Shear modulus(Pa)	7.99 × 10^10^
	Density(Kg/m^3^)	7800
Soil-Soil	Coefficient of restitution	0.532
	Static friction system	0.25
	Dynamic friction coefficient	0.4
Soil-Disk(60Si2Mn)	Coefficient of restitution	0.3
	Static friction system	0.5
	Dynamic friction coefficient	0.01

#### Establishment and analysis of tool motion equation

**Figure 2:**
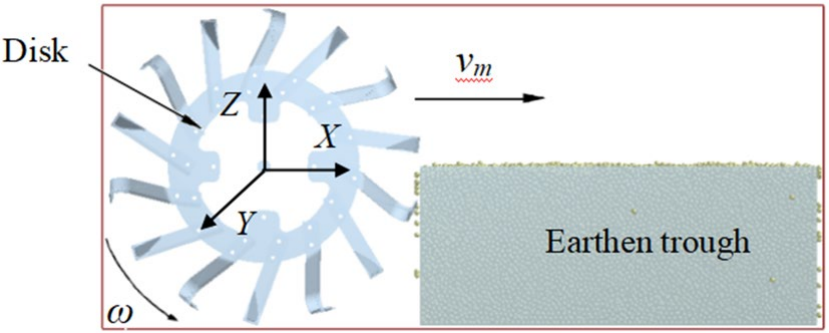
Soil-Disk operation model.

During the operation of the trenching device, the absolute motion of the tool is composed of the rotary motion of the cutter head and the forward motion of the trenching machine. When a complete set of trenching operations is completed, its motion path is a cycloid^33^.In addition to the physical characteristics of the soil particles, the factors that affect the soil cutting by the trenching cutter head in this movement process should also include the rotation speed of the cutter head, the positive and negative rotation speed and the forward speed of the device. In order to study the movement state of the trenching operation, according to the previous research results and the research basis^34^, the EDEM2020 software is used to establish the soil-cutter head interaction model of the coordinate system with the cutter head rotation center as the origin (as shown in Fig. 2). The forward speed and rotational speed of the cutter head are defined consistent with the test.

**Figure 3:**
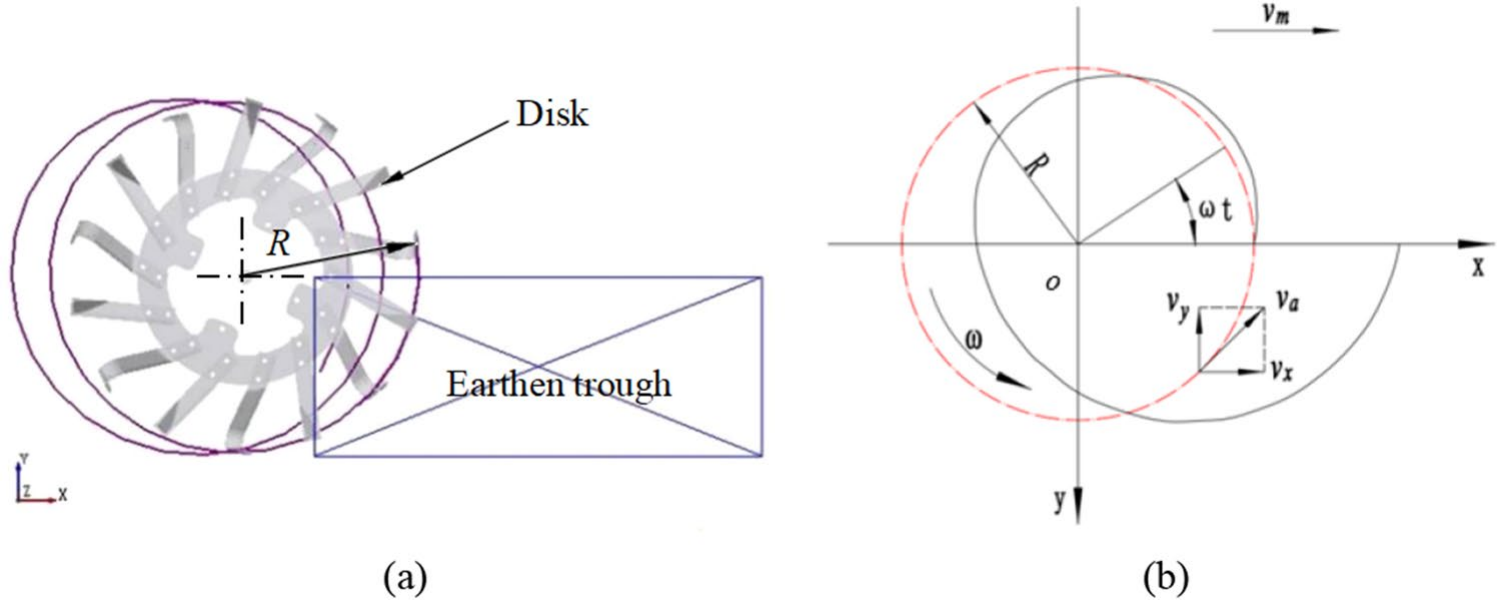
The movement path of the cutting point of the grooving tool. (**a**) Particle motion path at cutter head radius *R*; (**b**) Motion trajectory equation of trenching device.

In order to analyze the trenching process, the Manual Selection function of EDEM software is used, selecting a soil particle on any tool with the radius *R* of the cutter (as shown in Fig. 3a) (when the soil groove is cut, most of the soil particles are thrown out along the direction of the tool motion, and the relative motion between the particles and the cutter, the relative motion between the particles and the particles, the soil groove boundary is prone to rebound phenomenon, so the soil particle is fixed on the tool), the extraction particle trajectory is shown in Fig. 3a.The movement process is analyzed, and the movement mode of the trenching device during cutting operation is as follows. The movement mode of the trenching device is composed of the angular velocity *ω* of the trenching tool rotating around the cutter head center *O* and the trenching machine following the tractor with the Velocity *v*_m_. According to the extracted particle (point a) motion locus, it can be seen that the motion locus conforms to the equation of rake tool rotating motion locus, its motion path depends on the ratio of the circumferential velocity *v*_p_ = *Rω* of the cutter end point to the forward velocity *v*_m_ of the tractor. The motion trajectory equation of the trenching device is established as the ditch speed ratio is *λ* = *v*_p_ × *v*_m_^−1^ = *Rω* × *v*_m_^−1^ ( in which the horizontal velocity of the end point of the trenching device is opposite to the forward direction when the cutter rotates at a certain position, so that only when the cutter rotates at a certain position, the motion trajectory is a long cycloid, and the phenomenon of cocycloid winding can occur, thus the cutting edge of the trenching tool can cut the soil backward, and the lower rotation speed of the cutter can obtain a higher cutting speed). The forward direction of the trenching device is the positive direction of the *x-*axis of the coordinate system, and the vertical downward direction is the positive direction of the *y*-axis, as shown in Fig. 3b.

The motion trajectory is analyzed and the x-and y-direction coordinate equations are as follows:

1 $$\left\{ {\begin{array}{*{20}l} {x = v_{m} t + R\cos \omega t} \hfill \\ {y = R\sin \omega t} \hfill \\ \end{array} } \right.$$

In the equations, *R* is groove cutter head end turning radius, m;* t* is time, s; *v*_m_ is furrow machine forward speed, km/h; *ω* is cutter shaft angular speed, rad/s.

The speed of the tool end point at any time is analyzed under the condition of ignoring the friction and boundary conditions. Based on the motion path equation of the tool end point along the trenching device, the tangential speed of x-axis of the tool end point at any time is *v*_x_ and the normal speed of the y-axis is *v*_y_ :

2 $$\left\{ {\begin{array}{*{20}l} {v_{x} = \frac{dx}{{dt}} = v_{m} (1 - \lambda \sin \omega t)} \hfill \\ {v_{y} = \frac{dy}{{dt}}R\omega \cos \omega t} \hfill \\ \end{array} } \right.$$

At this time, the absolute speed of the tool end point is:

3 $$v = \sqrt {v_{m}^{2} + R^{2} \omega^{2} + 2v_{m} R\omega \sin \omega t}$$

#### Analysis of the influence of pitch of trenching and soil cutting

Through the construction of the motion trajectory equation of the trenching device, it can be seen that the main factors affecting the trenching performance are* R* (turning radius of the end of the trenching cutter head), *v*_*m*_ (forward speed of the trenching machine), *ω* (cutter shaft angular speed,* ω* = 2π*n*). Based on the agronomic requirement that the depth of trenching should not be less than 400 mm when applying organic fertilizer to fruit, in this study, the design turning radius *R* is 600 mm, and the rotation direction of the cutter head is opposite to the forward direction, and the trenching speed ratio is selected *λ.* The horizontal and longitudinal thickness of the soil layer—cutting pitch *S*, the size of the cutting pitch directly affects the quality of the broken soil and the flatness of the bottom. The calculation formula of the cutting pitch is as follows:

4 $$S = 6000v_{m} /nz = \pi R/5\lambda z$$

In the formula, *v*_*m*_ is unit forward speed (m/s);*R* is rotary blade radius (mm);*n* is rotary blade shaft speed (rad/min);*Z* is number of tools.

**Figure 4:**
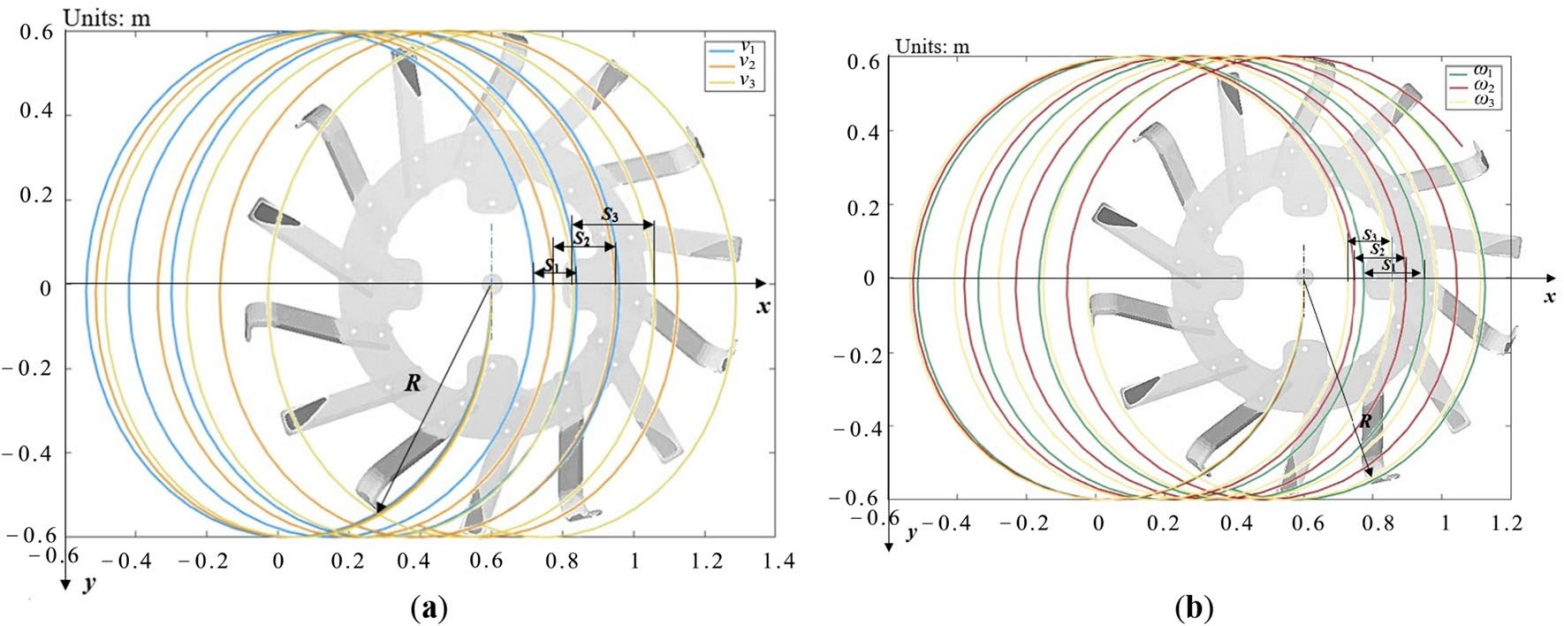
Trenching tool path and soil-cutting pitch with different motion parameter.

According to the above formula, under the same structural parameters, the radius of gyration *R* and the number of tools *Z* are fixed values, and different values of *v*_*m*_ and *ω* are selected (according to the actual situation of orchard trenching field operation in Xinjiang, *v*_*m*_ is set to 800 m/h, 1150 m/h and 1500 m/h; ω is 110 rad/min, 130 rad/min and 150 rad/min) for counter-rotation operation. The grooving tool motion trajectory and soil cutting pitch with different motion parameters drawn by Matlab, as is shown in Fig. 4, the forward direction of the trenching device in the figure is the positive direction of the *x*-axis of the coordinate system, and the vertical downward direction is the positive direction of the *y*-axis.

From Fig. 4, the trenching and cutting pitch *S* and the trajectory change with the increase and decrease of the different motion parameters (unit forward speed, knife shaft speed). Figure 4a shows that the fixed cutter shaft speed increases with the unit advance speed *v*_*m*_ at the same time and the trenching pitch increases accordingly. Figure 4b shows that the cutting pitch decreases as the knife shaft speed increases with the fixed unit speed. The kinematic and kinetic analysis of trenching shows that, within the range of tractor rated tractive force and power output torque, increasing the unit speed and cutter shaft speed increases tractive force and trenching tool cutting force, improves soil quality and reduces energy consumption from soil breaking.

#### The power consumption analysis of the trenching device

In this paper, the trenching tools are arranged in the form of successively spaced spiral. The power consumption of the trenching device is analyzed by the unit method in the process of the trenching device cutting the soil. In addition, the power consumption generated by the soil cutting process of the trencher is converted into the energy generated by the scattering of the cut soil particles^35^. Element method tell that the power consumption of rotary trenching device mainly consists of four parts: cutting power *W*_*Q*_, dispersing power *W*_*P*_, friction power *W*_*f*_ of collapsing soil and cutter head, and traction power *W*_*T.*_ Because the cutter head of rotary cutting does not produce the friction force caused by the gravity radial component and the gravity tangential component, the friction power consumption of the collapsed soil and the cutter head as well as the traction power consumption can be removed during the analysis process, the power consumption of the trenching tool is as follows:

5 $$W = W_{M} + W_{P} = \int {\rho \omega^{2} HB^{\prime } v_{m} \left( {\frac{R - H}{2}} \right)}^{2} dt + \int {\frac{\gamma }{2g}v_{0}^{2} \omega BSR\sin \sigma dt}$$

In the formula, *ρ* is soil density, kg/m^3^;*H* is trench depth, m; *B* is working width, m; *v*_*m*_ is unit forward speed, m/s; *γ* is soil bulk density, kg/m^3^; *g* is gravitational acceleration, 9.8 m/s^2^; *v*_*0*_ is the velocity at which the soil particle just leaves the cutter, m/s; *S* is the pitch of the soil cut, m, *S* = *v*_m_*dt*; *σ* is the starting angle of the soil slice in the cutter cutting unit, °; *dS* is the length of the soil unit, m, *dS*≈*Rdσ*.

In China Soil Database and Rotary Trencher, soil density of heavy loam *ρ* = 2.65 × 10^3^ kg/m^3^, soil bulk density *γ* = (1.0 ~ 1.5) × 10^3^ kg/m^3^. According to the following text, set the trenching cutter head to take the cutter head speed n and the unit forward speed v as the variables; working width of trenching device *B* = 0.3 m, turning radius of cutter head *R* = 0.6 m, gravity acceleration *g* = 9.8 m/s^2^, where *ω* = 2π*n*, *S* = *v*_m_*dt*. Substitute the known value into the formula, use the int function of Matlab to transform and program the formula (5), Through derived values, a conclusion can be got: the power consumption of trenching will increase with the increase of angular speed and forward speed.

### Trenching device field performance test

#### Test conditions and equipment

**Figure 5:**
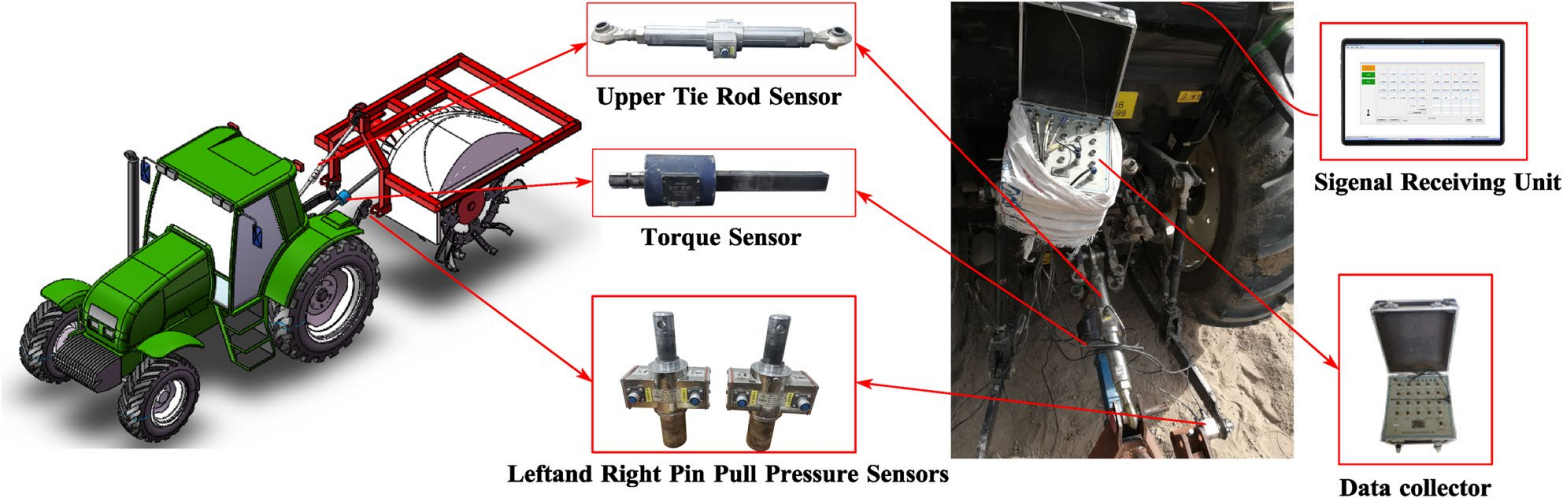
Installation diagram of NJTY3 general dynamic telemetry instrument for agricultural machinery.

The test have been carried out in Wugong village, Shihezi, Xinjiang, China (44°30′58′′N、86°04′10′′E) in July 2022. With a temperate continental climate, the author select the fruit tree seedling base with the same soil physical characteristics and less difference as the forest fruit planting as the test field. The average soil compactness is 2.16 MPa at the soil depth of 400 mm by SC900 soil compactness meter (SPECTRUM system, accuracy ± 1.25 cm, ± 15 PSI (103 kPa), measuring range 0 to 45 cm, 0 to 1000 PSI (0 to 7000 kPa)). The average soil moisture is 10.64% by TDR300 Soil Moisture meter (SPECTRUM, accuracy: ± 3.0 per cent, measuring range: 0—saturated (volumetric water content)). The test soil is gravel lime desert loess. The texture of 0 ~ 500 mm soil layer is heavy soil, massive structure, larger firmness. The test area is 1.4hm^2^. The matching power adopts TN654 tractor. The test equipment includes: trenching device, mechanical tachometer (made by Shanghai tachometer factory, precision ± 0.1%, measuring range 0 ~ 400r-min^−1^), tape measure (made by Hong Kong bunt bao industry, measuring range 0-50 m), NJTY3 general dynamic telemetry system (made by Heilongjiang Academy of Agricultural Machinery Engineering Science and technology, precision ± 0.05%, in which the torque and power consumption are measured by wireless telemetry technology, and the technical scheme of integrated torque sensor of power output shaft and traction sensor with three-point suspension without frame is used to measure the dynamic signals of trenching device, such as traction force and torque, and obtain test data), the installation diagram of NJTY3 general dynamic telemeter for agricultural machinery is shown in Fig. 5. The measuring method and index parameters were selected according to the experimental indexes stipulated in the agricultural industry standard “Field trenching machinery quality.” (NY/T 740 -2003), which were as follows: the power consumption of trenching operation, the depth of trenching, the width of trenching and the thickness of floating soil at the bottom of trenching.

#### Determination of trenching test and test plan

Each group of trenching test selects two operation strokes, and each stroke length is 10 m, and 5 points are measured at equal intervals, a total of 10 points are measured. Before the measurement, removing the topsoil at the bottom of the ditch, and placing a ruler between the original ground surface and the two ditch walls. The distance between the center point of the ditch bottom and the ruler is the trench depth, trench width and the thickness of the topsoil at the bottom of the ditch. Measuring multiple times of the test points. In order to study the change rule of different structural parameters and working parameters on the trenching performance, through the analysis of the actual working environment and influencing factors, the test selects the forward speed, cutter head speed and different tool combinations as the inspection factors, and takes the trenching power consumption, trenching depth, trenching width, and topsoil thickness as the evaluation indicators of the trenching performance, and analyzes the impact of different tool combinations and working parameters of the trenching device on the trenching quality. During trenching operation, in order to give full play to the power of the tractor, setting the forward speed of the unit as 800 m/h, 1150 m/h and 1500 m/h according to the actual working requirements of trenching operation and the soil conditions in Xinjiang.

According to Section (Analysis of The Influence of Pitch of Trenching and Soil Cutting) of this paper, we can see the impact of the cutter head rotation speed on the trenching operation. This test adopts the reverse milling method, that is, the cutter head rotation direction is opposite to the unit forward direction, the trenching tools put into the soil from the ground, and starts from the bottom of the soil to be cut, and rotates counterclockwise to cut the soil and throw the soil up. According to the actual situation of Xinjiang orchard trenching fertilization, the three horizontal values of the cutter head rotation speed are set as 110 r/min,130 r/min, 150 r/ min.

According to the soil characteristics, fruit planting mode and fertilization requirements in Xinjiang, the three factors, affecting the trenching operation performance, are determined from the determination of Section (Analysis of The Influence of Pitch of Trenching and Soil Cutting) factors, affecting the trenching performance, the power consumption analysis of Section (The Power Consumption Analysis of The Trenching Device) trenching device, and the agronomic requirements and actual production level of orchard trenching fertilization. The three factors are set at three levels respectively for field test room analysis. The factor level table is shown in Table 2, and the tool structure parameters are shown in Table 3:

**Table 2:** Factor level table.

Levels	Factors		
	Forward speed v/(m·h^−1^)	Rotary speed of cutter head n/(r·mim^−1^)	Cutter head combination
-1	800	110	A
0	1150	130	B
1	1500	150	C

**Table 3:** Tool structure parameters.

Cutter head combination	A	B	C
Earth cutting angle *β*/(°)	130	140	150
Bending angle *α* /(°)	40	50	60
wide working range of Scimitar* b*/(mm)	182	156	133
Earth cutting angle *β*/(°)	180	180	180
Bending angle *α* /(°)	90	90	90
Wide working range of Scimitar *b*/(mm)	117.5	143	166

### Research involving plants

The experimental studies and and field research in this paper did not involve any plants (either cultivated or wild), nor any endangered plants.

## Results

### Test results and analysis of trenching device

#### Establishment of regression model and analysis of variance

In order to verify and optimize the combination and working parameters of trenching cutter head, response surface test method is adopted. The field test is carried out with the influencing factors of the advance speed of the unit, the rotating speed of the cutter head and the combination of the cutter head, and the test indexes of the power consumption, the depth of the ditch, the width of the ditch and the thickness of the floating soil at the bottom of the ditch. The test protocol and results are shown in Table 4.

**Table 4:** The process and results of experiment.

Test No	Factors			Test indicators			
	Advance speed *X*_1_/(m·h^−1^)	Rotating speed of the cutter head *X*_2_/(r·min^−1^)	Combination of the cutter head/*X*^3^	The depth of the ditch*Y*_1_/(mm)	The width of the ditch*Y*_2_/(mm)	Thickness of the floating soil at the bottom *Y*_3_/(mm)	Power consumption*Y*_4_/(kW)
1	− 1	− 1	0	324	388	94	19.54
2	1	− 1	0	318	328	108	25.55
3	− 1	1	0	328	340	140	26.46
4	1	1	0	392	352	62	36.76
5	− 1	0	− 1	384	340	34	27.63
6	1	0	− 1	354	328	46	44.97
7	− 1	0	1	350	348	86	40.74
8	1	0	1	416	372	104	50.64
9	0	− 1	− 1	360	336	58	25.75
10	0	1	− 1	338	326	66	34.03
11	0	− 1	1	400	330	98	37.47
12	0	1	1	384	340	52	29.96
13	0	0	0	280	318.8	54	28.09
14	0	0	0	240	305	30	32.65
15	0	0	0	250	310	40	37.31
16	0	0	0	200	300	20	31.44
17	0	0	0	230	310	25	33.43

Using Design-Expert 13.0.1.0 to fit and analyzing variance of the test results in Table 4, the regression model of ditch depth, ditch width, depth of floating soil at the bottom of ditch, and power consumption of actual operation can be obtained, as shown in Eqs. (6), (7), (8), (9).

6 $$\begin{aligned} Y_{1} = & 240 + 15.71X_{1} + 1.04X_{2} - 14.25X_{3} - 64.75X_{1} X_{2} \\ - & 24X_{1} X_{2} - 1.50X_{2} X_{3} + 96.04_{1}^{2} + 86.71X_{2}^{2} + 41.87X_{3}^{2} \\ \end{aligned}$$

7 $$\begin{aligned} Y_{2} = & 308.76 - 2.33X_{1} - 5.17X_{2} - 7.50X_{3} + 18.26X_{1} X_{2} \\ - & 9X_{1} X_{3} - 5X_{2} X_{3} + 28.57_{1}^{2} + 14.41X_{2}^{2} - 51.50X_{3}^{2} \\ \end{aligned}$$

8 $$\begin{aligned} Y_{3} = & 33.80 - 0.17X_{1} - 8.83X_{2} - 17.00X_{3} - 127.2X_{1} X_{2} \\ - & 1.50X_{1} X_{3} - 13.50X_{2} X_{3} + 89.97_{1}^{2} + 82.03X_{2}^{2} - 51.50X_{3}^{2} \\ \end{aligned}$$

9 $$\begin{aligned} Y_{4} = & 32.58 + 7.35X_{1} + 0.46X_{2} - 3.30X_{3} + 0.92X_{1} X_{2} \\ + & 1.86X_{1} X_{3} + 13.95X_{2} X_{3} + 1.78_{1}^{2} - 7.13X_{2}^{2} + 6.49X_{3}^{2} \\ \end{aligned}$$

By using the Design-expert software to analyze the test data, the regression model variance analysis tables of the test factors on the ditch depth, the ditch width, the bottom floating soil thickness and the actual operation power consumption are obtained, as shown in Table 5.

**Table 5:** Analysis of variance of variance.

Indexes	Ditch depth			Ditch width			Depth of floating soil at the bottom of the ditch			Operation power consumption		
Source of variation	*F* value	*p* value	Significance	*F* value	*p* value	Significance	*F* value	*p* value	Significance	*F* value	*p* value	Significance
Model	9.38	0.0037	**	4.67	0.0272	*	4.67	0.0272	*	11.03	0.0023	**
*X*_1_	2.00	0.2004	–	4.419E-004	0.9838	–	4.419E-004	0.9838	–	35.71	0.0006	**
*X*_2_	0.008786	0.9279	–	1.24	0.3020	–	1.24	0.3020	–	0.14	0.7220	–
*X*_3_	2.19	0.1823	–	6.13	0.0425	*	6.13	0.0425	*	9.62	0.0173	*
*X*_1_*X*_2_	1.92	0.2086	–	14.54	0.0066	–	14.54	0.0066	–	0.031	0.8643	–
*X*_1_*X*_3_	3.11	0.1212	–	0.024	0.8816	–	0.024	0.8816	–	1.52	0.2568	–
*X*_2_*X*_3_	0.012	0.9153	–	1.93	0.2070	–	1.93	0.2070	–	6.87	0.0344	*
*X*_1_^2^	14.36	0.0068	**	24.43	0.0017	**	24.43	0.0017	**	0.40	0.5455	–
*X*_2_^2^	11.71	0.0111	*	20.59	0.0027	**	20.59	0.0027	**	6.47	0.0385	*
*X*_3_^2^	3.44	0.1059	–	10.23	0.0151	*	10.23	0.0151	*	6.75	0.0355	*
Residual error			–			–			–			–
Missing items	0.70	0.5989	–	3.50	0.1290	–	3.50	0.1290	–	0.57	0.6648	–

*P* ≤ 0.01 is very significant, marked as **; 0.01 < *p* ≤ 0.05 is significant, marked as *; *P* > 0.05 is not significant, marked as-.

From the analysis of variance in Table 5, the regression model of trench depth and operation power consumption is very significant, the regression model of trench width and bottom floating soil thickness is significant, while. The results show that the fitted regression equations of the model of trench depth, trench width, depth of floating soil at the bottom of the trench and working power consumption are in good agreement with the actual situation. It can predict the relationship between the depth of ditch, width of ditch, the thickness of floating soil at the bottom of ditch, operation power consumption and each test factor. The *P* values in the table can be as follows: (1) the square term of the advance speed* X*_1_^2^ has a significant effect on the ditch depth, and the square term of the rotary speed of the cutter head *X*_2_^2^ has a significant effect on the ditch depth. The effect of advance speed, rotary speed of cutter head and combination of cutter head on ditch depth is not significant. According to the regression equation of ditch depth, the square term *X*_2_^2^ is the main factor that affects the ditch depth. (2) the square term of advance velocity *X*_1_^2^ has a significant effect on the trenching depth, while advance velocity, rotary speed of cutter head and combination of cutter head have no significant effect on the trenching depth, being combined with the ditch depth regression equation, the results show that the main factors affecting the width of ditch are the square term of advance velocity *X*_1_^2^. (3) the square term of advance velocity *X*_1_^2^ and the square term of cutter head rotational speed *X*_2_ have a significant influence on the thickness of soil return at the bottom of ditch, the influence of square term *X*_3_^2^ of cutter head combination *X*_3_ and cutter head combination is significant. According to the regression equation of the thickness of the floating soil at the bottom of the gully, the factors influencing the thickness of the floating soil at the bottom of the gully are the square term of the advancing speed *X*_1_^2^, the square term of the rotating speed of the cutter head *X*_2_^2^ and the square term of the cutter head combination *X*_3_^2^ (4) the forward speed *X*_1_ has a significant effect on the actual operation power consumption, the interaction between Cutter Head Assembly *X*_3_, Cutter head rotation speed *X*_*2*_ and Cutter head assembly *X*_3_, the square term of cutter head rotation speed *X*_2_^2^ and the square term of cutter head assembly *X*_3_^2^ are significant. Combined with the power consumption regression equation of actual operation, through the analysis, we can know that the main factors affecting the power consumption are the forward speed *X*_*1*_, the cutter head combination *X*_3_, the interaction between the cutter head speed *X*_2_ and the cutter head speed *X*_3_, the cutter head speed square term *X*_2_^2^, the cutter head combination square term *X*_3_^2^. According to the analysis of variance for each factor, after excluding non-significant factors, the relationship is as follows:

10 $$Y_{1} = 240 + 86.71X_{2}^{2}$$

11 $$Y_{2} = 308.76 + 28.57X_{1}^{2}$$

12 $$Y_{3} = 33.80 - 17.00X_{3} + 89.97X_{1}^{2} + 82.03X_{2}^{2} - 51.50X_{3}^{2}$$

13 $$Y_{4} = 32.58 + 7.35X_{1} - 3.30X_{3} + 3.95X_{2} X_{3} - 7.13X_{2}^{2} + 6.49_{3}^{2}$$

#### Response surface analysis

**Figure 6:**
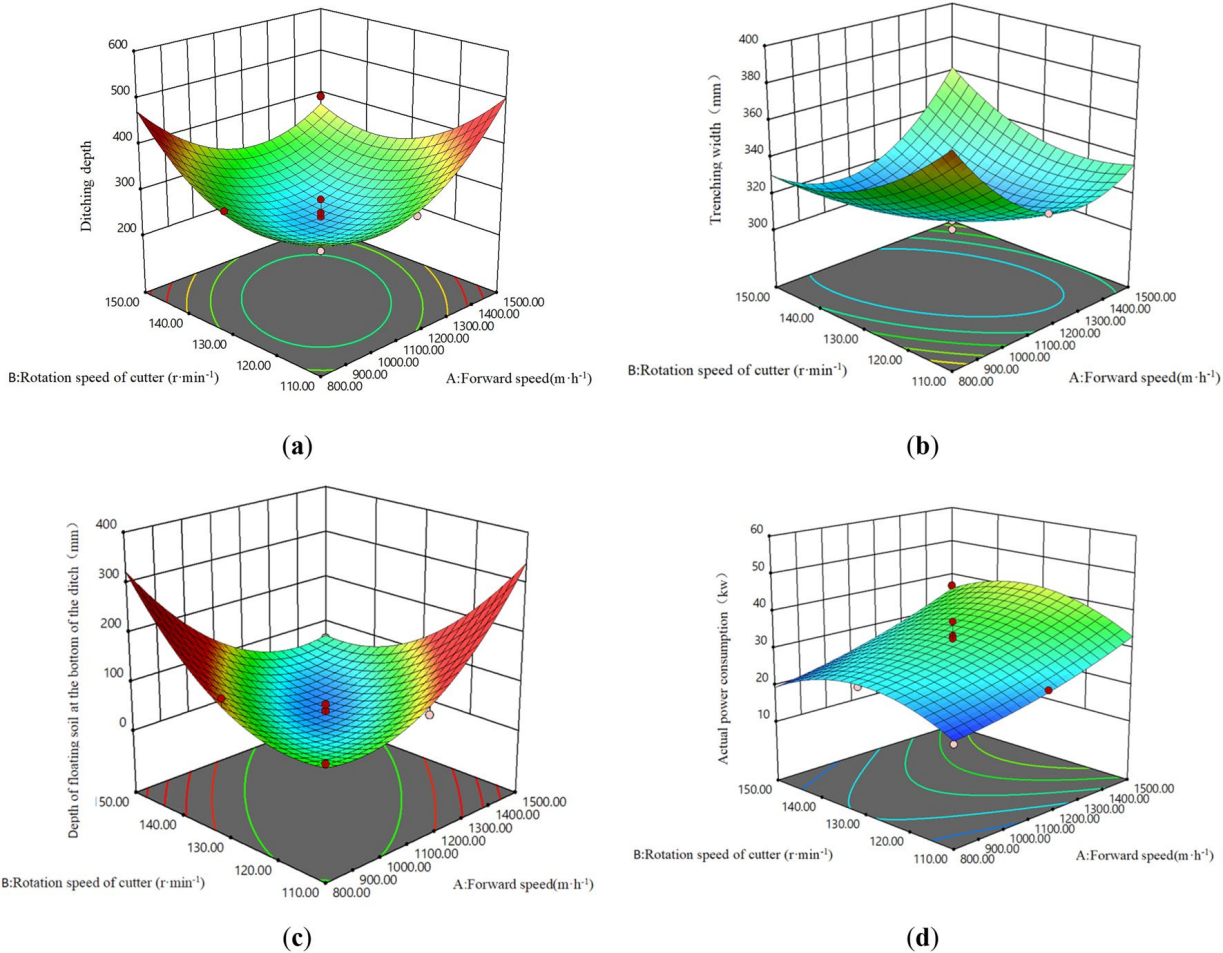
Trenching performance response surface. (**a**) Response surface of trench depth; (**b**) Response surface of ditch width; (**c**) Response surface of the thickness of the backsoil at the bottom of the ditch; (**d**) Response surface of the actual operation power consumption.

Using Perturbation function of Design-Expert software, the response surface analysis of the relationship between the advance speed *X*_1_, the cutter head speed *X*_2_, the cutter head combination *X*_3_ and the trench depth, the trench width, the depth of the bottom floating soil, and the actual power consumption is carried out, as shown in Fig. 6.

The response surface based on the trenching performance response surface regression equation is shown in Fig. 6. From Fig. 6, the trenching quality is closely related to the cutter head combination, the cutter head rotating speed and the forward speed. When the cutter head combination is B, with the increase of the cutter head speed and forward speed, the trench depth, trench width and the thickness of the floating soil at the bottom of the ditch decrease first and then increase, and the power consumption of the trench operation increase gradually. When the rotating speed of the cutter is 130r/min and the advancing speed is 1150 m/h, the furrow depth, the furrow width and the thickness of the floating soil at the bottom of the furrow reach the minimum, and the rotating speed of the cutter and the advancing speed have the greatest influence on the power consumption of the furrow operation. As is shown in Fig. 6a, the furrow depth gradually decrease with the current advancing speed 800 < *X*_1_ < 1150 m/h, the cutter head speed 110 < *X*_2_ < 130r/min; while the furrow depth gradually increase with the current advancing speed 1150 < *X*_1_ < 1500 m/h, the cutter head speed 130 < *X*_2_ < 150r/min. As is shown in Fig. 6b, the trench width decreases with increasing forward speed when the cutter speed is 110 < *X*_2_ < 130r/min, and increases with increasing forward speed when the cutter speed is 130 < *X*_2_ < 150r/min. As shown in Fig. 6c, when the forward speed is low, the thickness of the floating soil at the bottom of the ditch increases with the increase of the rotating speed of the cutter head. As is shown in Fig. 6d, when the cutter speed is less than 130r/min, the influence of the cutter speed on the power consumption of trenching operation is greater, while when the cutter speed is more than 130r/min and the advancing speed is more than 1150 m/h, the influence of the cutter speed on the power consumption of trenching operation is smaller. When the current forward speed is 800 < *X*_1_ < 1150 m/h, the cutter head speed is 110 < *X*_2_ < 130r/min, the power consumption of trenching operation is low.

### Parameter optimization and validation experiment

#### Establish the optimization model

The trenching quality is the main basis to determine the structure parameters and working parameters of the trenching device for deep application of organic fertilizer in fruit in Xinjiang. In order to get the best level of experimental factors, according to the characteristics of the furrow with organic fertilizer (furrow depth should not be less than 400 mm, furrow width should not be less than 250 mm) and the quality index of the field furrow machinery, the furrow depth *H* = (1 ± 0.1)*H*, furrow width *B* = *B* ± 1.5, the thickness of the bottom of the ditch is less than 0.1*H*, the working power consumption should be as low as possible to meet the working index of the trenching machine. Combined with the boundary conditions of test factors, the mathematical model of parameter optimization of advance speed, cutter head speed and cutter head combination is established. And the regression model is analyzed according to the boundary conditions of experimental factors to get the mathematical model:

14 $$\left\{ {\begin{array}{*{20}l} {Y_{1} \ge 360,Y_{2} \ge 250,Y_{3} \le 36,18 \le Y_{4} \le 50} \hfill \\ {s.t.\left\{ {\begin{array}{*{20}l} {X_{1} \in [800,1500]} \hfill \\ {X_{2} \in [100,150]} \hfill \\ {X_{3} \in [ - 1,1]} \hfill \\ \end{array} } \right.} \hfill \\ \end{array} } \right.$$

In the formula, *Y*_1_, *Y*_2_, *Y*_3_ and *Y*_4_ are the target functions of trench depth, trench width, bottom floating soil thickness, and trench power consumption, respectively; *X*_1_ is the forward speed, m/h; *X*_2_ is the cutter speed, r/min; *X*_3_ is the cutter head combination. The Optimization function provided by Design-Expert software is used to optimize the response surface test data, and the optimal parameter combination is obtained as follows: the forward speed is 803 m/h, the cutter head speed is 111r/min, the combination of cutter head is C (cutter A cutting angle 150°, bending angle 60°, working width 133 mm; cutter B cutting angle 180°, bending angle 90°, working width 166 mm), the furrow depth is 382 mm, the furrow width is 288 mm, the thickness of the floating soil in the bottom of the ditch is 8 mm, the power consumption of the trenching operation is 18.91 KW, and all the working indexes have reached the technical requirements of the relevant national standards.

#### The application of the optimization model

In order to verify the reliability of the optimization results, field validation tests are carried out. Select 50 m flat test area, set up trench operation forward speed of 803 m/h, the cutter head speed of 111r/min, the cutter head is composed of C (cutter A cutting angle 150°, bending angle 60°, working width 133 mm; cutter B cutting angle 180°, bending angle 90°, working width 166 mm). The test is repeated 3 times each time, and the power consumption data are collected by the general dynamic telemetry system of agricultural machinery, and the average values of gully depth, gully width and floating soil thickness are collected from 5 measuring points.

According to Table 6, the average depth of ditch is 472 mm, the width is 332 mm, the thickness of floating soil is 134 mm, and the power consumption of ditch operation is 19.95 KW. All work indicators have exceeded the technical requirements of relevant national standards. It is shown that the proposed optimization model has good engineering practicability and high accuracy for obtaining the performance values of actual trenching operations, it also provides a theoretical reference for selecting the optimal parameter combination of the sectional cutting trenching device which can save power and satisfy the trenching Operation Performance Index.

**Table 6:** Field test results.

Field test values				
Numbers	Depth of the ditch *Y*_1_/(mm)	Width of the ditch *Y*_2_/(mm)	Thickness of the floating soil at the bottom *Y*_3_/(mm)	Power consumption* Y*_4_/(kW)
1	450	360	15	20.45
2	440	380	14	19.71
3	490	310	16	20.24
4	410	320	10	19.55
5	550	300	15	19.83
Average	472	332	13	19.95

## Discussion

The purpose of this paper is to study the influence law of disc-type trenching device in orchards, using a combination of theoretical analysis and field experiments to explore the influence law of motion trajectory, performance and quality of trenching and cutting operation. To explore the trend of interaction effects among factors, the response surface method is used to design field experiments. In order to maximize the demand of fertilizer application agronomy in orchards, an optimization model is constructed with the objective of optimal trenching performance and quality, and the accuracy of the optimization model is verified.

Variations in furrowing operating parameters, which is influenced by the depth of furrowing, rotational speed of the cutter disk, and structural parameters, have a significant effect on furrowing performance and quality^19, 23, 24^. For example, Ye Qiang et al.^36^ conducted field performance tests on the furrowing device to improve the furrowing efficiency of vineyard furrowers in Hunan. The tests showed that the device can reduce power consumption and machine quality in two operations, and can achieve the agronomic requirements of furrowing depth and width, etc.. But the model is only applicable to the dwarf planting pattern in southern China. To solve the problems, such as poor uniformity of furrowing depth and high operational resistance on the no-till planter in northeast China, Zhao Shuhong et al.^37^ designed a segmented corn furrowing and seeding device. Through field comparison tests between the segmented furrowing device and other different furrowing devices, they also conducted field performance tests on the no-till seeder for deep fertilization of corn in northeast China. It was concluded that the segmented furrowing device has advantages such as high uniformity of furrowing depth, low soil disturbance, and low operational resistance, and no parameter search was conducted. However, there are relatively few studies on trenching based on the agronomic requirements of organic fertilizer application in Xinjiang orchards, which may lead to a certain degree of influence on its trenching effect.

To study the influence law of trenching operation parameters on trenching quality, the response surface method is used to design the field experiment. The results of ANOVA showed that the regression model of ditching depth and operating power consumption is very significant, while the regression model of ditching width and ditching bottom floating soil thickness is significant, but the misfit is not significant. The established regression model of each index is consistent with the actual situation and can predict the relationship between each index and experimental factors. Based on the response surface analysis of the relationship between the parameters, the trenching depth, width and floating soil thickness decrease first and then increase with the increase of rotational speed and forward speed, while the operating power consumption increases gradually.

The results of the optimisation tests meet the quality performance indicators of the deep application of organic fertiliser in Xinjiang orchards. However, there are some problems. In order to reduce the amount of software calculations, the influence of deflectors, bolts and screws on the simulation is simplified, leading to more serious soil backflow during the simulation; on the other hand, the influence on the frame, deflectors and drive machine in the theoretical analysis of the trenching operation is ignored. At the same time, in the field operation process, there is inevitably friction and wear of roots, film residues and stones, etc., resulting in additional power consumption, so the power consumption value of the field test operation is relatively higher than the theoretical value.

## Conclusions

According to the characteristics of applying organic fertilizer to fruit in Xinjiang -northwest China, theoretical analysis and experimental study has been carried out with the aim of affecting the performance of disc trenching device, the conclusions are as follows:

According to the requirements of deep application of organic fertilizer, planting patterns and physical characteristics of soil in Xinjiang, the effects of different combinations of cutter head on the movement trajectory are analyzed; the effects of the combination of the cutter head, the rotating speed of the cutter head and the advancing speed on the trenching performance are studied.

The field experiment is conducted to analyze the effects of different tool combinations on trenching quality (width, depth, thickness of floating soil at the bottom of ditches) and power consumption. The test results show that with the increase of cutter speed and advance speed, the trench depth, the trench width and the thickness of the floating soil at the bottom of the trench decrease first and then increase, while the power consumption of the trench operation increases gradually.

Taking the best trenching quality as the optimization objective, an optimization model of trenching operation quality is established. The optimal parameters of trenching operation quality are as follows: the advance speed is 803 m/h, the rotating speed of cutter head is 111 r/min, and the cutter head combination C (two cutters are a group, which are arranged spirally at intervals, the cutting angle of cutter is 150°, the bending angle is 60°, and the working width is 133 mm; the cutting angle of Cutter B is180°, the bending angle is 90°, and the working width is 166 mm). The optimization test have shown that the average depth of trenching is 472 mm, the width is 332 mm, the thickness of back soil is 134 mm, and the working power consumption is 19.95 kW. In order to verify the accuracy of the optimized model and the field experiment, the validation experiment has been carried out, which meet the requirements of low energy consumption and deep trenching of forest fruits in Xinjiang, and have higher accuracy with the trenching quality optimization model and the experimental results.

## Data Availability

Data underlying the results presented in this paper are available from Prof. Wang Lihong(wlh_shz@163.com) upon reasonable request.
